# Epidemiological profile of kidney cancer in Brazil: a multiregional ecological study

**DOI:** 10.1590/2175-8239-JBN-2024-0180en

**Published:** 2025-03-10

**Authors:** Carlos Eduardo da Silva, Yasmin de Souza Ciriaco, Gustavo Machado Ribeiro, Laura Almeida Vidal, Verônica Aparecida Silva Cintra, Sabrina Thalita dos Reis

**Affiliations:** 1 Faculdade de Medicina Atenas Passos, Passos, MG, Brazil.; 2 Universidade de São Paulo, Faculdade de Medicina, Hospital das Clínicas, Laboratório de Investigação Médica 55, São Paulo, SP, Brazil.

**Keywords:** Kidney Neoplasms, Epidemiology, Hospitalization, Time Series Studies

## Abstract

**Introduction:**

Renal neoplasia is a complex and heterogeneous disease, characterized by high morbidity and mortality.

**Objective:**

To analyze the temporal trend of hospitalization rates (HRs) for renal neoplasia in Brazil, segmented by region, states (UFs, *Unidades da Federação* in Portuguese), and population characteristics, from 2013 to 2023.

**Methods:**

Ecological study using data from the Hospital Information System, by analyzing Hospital Admission Authorizations, covering the period from 2013 to 2023. The annual trend of HRs was analyzed using generalized linear regression with the Prais-Winsten method by calculating the Annual Percentage Change (APC), considering sex, age, race/color, and region/state (UF). A significance level of 5% was adopted for the analyses.

**Results:**

A total of 31,388 hospitalizations for renal neoplasia were recorded in Brazil during the period, showing a significant upward trend in HRs (APC: 9.12; 95%CI: 5.30; 13.1; p < 0.001). The increase was observed in both sexes and in all regions. Among the states, most showed stationary trends. The highest average HRs were identified among young elderly individuals (3.31/100,000) and long-lived elderly individuals (2.51/100,000).

**Conclusion:**

HRIs due to renal neoplasia in Brazil showed a significant upward trend between 2013 and 2023, with regional variations, a predominance in males, and a higher incidence in the over-60 age group.

## Introduction

Renal neoplasia is a complex and heterogeneous disease, comprising tumor subtypes that affect the kidneys, each with distinct histological alterations, genetic mutations, clinical characteristics, and responses to treatment^1,2^. Among these subtypes, renal cell carcinoma (RCC) is the most common, accounting for approximately 90% of all kidney tumors. Clear cell RCC is the most prevalent (70% of cases), followed by papillary RCC (10–15%), and chromophobe RCC (5%)^3^.

Renal neoplasia is more common in men than in women^4^, with a mean age at diagnosis of 65 years. It is strongly associated with risk factors such as smoking, obesity, systemic arterial hypertension, and genetic syndromes such as the von Hippel-Lindau syndrome^5,6,7^. From an epidemiological perspective, data from the Mortality Information System (SIM - *Sistema de Informação sobre Mortalidade*) recorded 18,306 deaths due to renal neoplasia in Brazil between 2018 and 2022^8^. This scenario reflects a process of population aging, associated with the prevalence of risk factors for the disease, highlighting the relevance of this disease as a public health concern^7^.

Renal neoplasia is initially asymptomatic, but may present with nonspecific symptoms such as flank pain, hematuria, palpable abdominal mass, fever, and anemia, hindering early diagnosis^9^. For this reason, the disease is often discovered incidentally^10^, through exams such as ultrasound, MRI, or abdominal CT scan and is subsequently confirmed by biopsy for histopathological classification: clear renal cell carcinoma (the most common and with the worst prognosis), papillary RCC (microscopically divided into type 1 - with a better prognosis - or type 2) and chromophobe RCC, which has the best prognosis when compared to clear cell RCC^11^.

This neoplasm is characterized by its high mortality rate, primarily due to its asymptomatic onset, lack of screening, and the difficulty in achieving early diagnosis. These factors contribute to the patient’s poor prognosis and death as a result of disease progression^10,12,13^. Given the growing relevance of renal neoplasia in the national epidemiological scenario, mapping regional distribution and hospitalization patterns over time is relevant. In view of this challenging scenario, the present study aims to fill this gap by contributing to a mapping of the epidemiology of renal neoplasia in Brazil, focusing on its geographic distribution, sociodemographic profile of patients, and temporal trends.

## Methods

This is an ecological, observational, quantitative study, analyzing hospitalization rates (HRs) due to the underlying cause of renal neoplasia, between 2013 and 2023, in Brazil, its regions, and states (UFs). Hospital Admission Authorizations (AIHs, in Portuguese) were analyzed and the variables sex, age group, Brazilian region and UF were studied.

The data were collected from the Information Technology Department of the Brazilian National Health System (DATASUS), using the Hospital Information System (SIH, in Portuguese), through the AIHs. The AIH is an essential tool for consolidating information about hospitalizations within the scope of the Brazilian National Health System, including detailed information on hospital care, such as diagnoses, procedures, length of stay, among other variables of interest for epidemiological and healthcare analysis. The underlying cause was defined by the C64 classification of the International Classification of Diseases 10th revision (ICD-10), referring to malignant kidney neoplasia, except renal pelvis.

The data were extracted and tabulated in Microsoft Excel® 2016, and subsequently, the categorical quantitative variables were analyzed in absolute and relative frequencies, presented in tables and graphs. The annual HRs for renal neoplasia were determined based on population estimates from the Brazilian Institute of Geography and Statistics (IBGE, in Portuguese) for the analyzed period, calculated by the ratio between the number of cases and the population size in each Brazilian region, multiplied by 100,000 inhabitants. The annual HRs were stratified by sex (male and female), age group (children and adolescents, up to 19 years old; young adults, 20 to 39 years old; adults, 40 to 59 years old; young elderly, 60 to 79 years old; long-lived elderly, 80 years or older) and region/UF (North [Rondônia, Acre, Amazonas, Roraima, Pará, Amapá, and Tocantins], Northeast [Maranhão, Piauí, Ceará, Rio Grande do Norte, Paraíba, Pernambuco, Alagoas, Sergipe, and Bahia], Southeast [São Paulo, Rio de Janeiro, Minas Gerais, and Espírito Santo], South [Paraná, Santa Catarina, and Rio Grande do Sul] and Central-West [Mato Grosso do Sul, Mato Grosso, Goiás, and the Federal District]). The graphical representation of renal neoplasia across the different Brazilian regions was created using Quantum GIS (QGIS) software, version 3.0.

Generalized linear regression using the Prais-Winsten method was employed to analyze the trend in HRs, as described by Antunes and Cardoso^14^. The coefficient’s annual percentage change (APC) was calculated using the logarithm of the coefficients as the dependent variable and the years of the historical series as the independent variable, adopting the number of years in the series –1. The trend was quantified by the equation: APC = [–1 + 10^b] * 100%, where “b” indicates the annual growth rate. Confidence intervals (95%CI) were calculated based on the formula: 95%CI = [–1 + 10^b±t*se] * 100%. The “b” values and the standard error (se) were extracted from the regression analysis, while the “t” value was obtained from the Student’s t distribution. A significance level of 5% (p < 0.05) was adopted, considering the upward trend when the rate was positive, downward when it was negative, and stationary when there was no statistically significant difference (p > 0.05). The analysis was conducted using the R Project for Statistical Computing software, version 3.6.0, with the assistance of Excel 2016 for database creation.

This study was based solely on public data made available by the Brazilian Ministry of Health and did not require approval from the Research Ethics Committee. The study complies with the guidelines established by Resolution No. 510 of 2016 of the Brazilian National Health Council and is in accordance with Law No. 12,527 of November 18, 2011.

## Results

Between 2013 and 2023, a total of 31,388 hospitalizations for renal neoplasia were recorded in Brazil. During this period, a significant upward trend was observed in HRs, rising from 0.48 per 100,000 inhabitants in 2013 to 2.62 per 100,000 inhabitants in 2023, with a mean HR of 1.35 per 100,000 inhabitants (APC: 9.12; 95%CI: 5.30; 13.1; p < 0.001) (Table 1). In both males and females, HRs showed a significant upward trend, with mean coefficients of 1.55 and 1.12 per 100,000 inhabitants, respectively (Table 2).

**Table 1:** Mean and trend of length of stay and hospitalization rate for renal neoplasia in the SUS by region and State, Brazil, 2013–2023

Region and State (UF)	HRs 2013	HRs 2023	Average HRs	APC (95%CI)	p-value	Trend
**Brazil**	**0.48**	**2.62**	**1.35**	**9.12 (5.30;13.1)**	**<0.001**	**Upward**
**North**	**0.30**	**1.40**	**0.73**	**7.61 (6.2;9.05)**	**<0.001**	**Upward**
Rondônia	0.18	0.29	0.63	4.95 (−0.35;10.55)	0.101	Stationary
Acre	0.75	0.25	0.37	3.42 (−5.28;12.95)	0.472	Stationary
Amazonas	0.35	0.39	0.78	6.56 (1.60;11.76)	0.028	Upward
Roraima	0.00	0.26	0.36	3.28 (−8.42;16.47)	0.612	Stationary
Pará	0.27	0.38	0.70	5.07 (1.29;8.99)	0.027	Upward
Amapá	0.54	0.23	0.30	−2.82 (−9.94;4.86)	0.480	Stationary
Tocantins	0.20	0.24	0.39	2.72 (1.32;4.15)	0.004	Upward
**Northeast**	**0.37**	**1.80**	**0.91**	**8.56 (5.49;11.71)**	**<0.001**	**Upward**
Maranhão	0.32	0.34	0.62	6.06 (3.25;8.95)	0.002	Upward
Piauí	0.62	0.61	1.05	4.95 (1.83;8.17)	0.012	Upward
Ceará	0.40	0.52	0.93	5.33 (−1.19;12.28)	0.146	Stationary
Rio Grande do Norte	0.42	0.68	1.36	7.02 (0.63;13.80)	0.059	Stationary
Paraíba	0.39	0.43	0.80	4.14 (−1.11;9.66)	0.158	Stationary
Pernambuco	0.47	0.42	0.72	3.26 (−1.29;8.02)	0.196	Stationary
Alagoas	0.34	0.36	0.72	6.51 (−2.31;16.11)	0.186	Stationary
Sergipe	0.23	0.49	0.87	5.89 (2.40;9.50)	0.009	Upward
Bahia	0.26	0.30	0.63	4.26 (−3.22;12.32)	0.300	Stationary
**Southeast**	**0.48**	**2.83**	**1.44**	**9.62 (5.28;14.14)**	**0.002**	**Upward**
Minas Gerais	0.47	0.69	1.46	5.30 (−2.84;14.12)	0.240	Stationary
Espírito Santo	0.54	0.58	1.09	5.26 (−0.94;11.85)	0.132	Stationary
Rio de Janeiro	0.43	0.39	0.78	3.58 (−1.87;9.34)	0.234	Stationary
São Paulo	0.50	0.66	1.32	4.53 (−3.47;13.19)	0.304	Stationary
**South**	**0.82**	**4.38**	**2.33**	**9,15 (4,99;13,48)**	**0.002**	**Upward**
Paraná	0.96	1.12	2.16	3.91 (−2.99;11.31)	0.302	Stationary
Santa Catarina	0.48	0.68	1.44	4.93 (−2.27;12.66)	0.217	Stationary
Rio Grande do Sul	0.88	1.19	2.27	4.97 (−2.58;13.11)	0.235	Stationary
**Central-West**	**0.51**	**2.47**	**1.30**	**8.42 (4.34;12.67)**	**0.003**	**Upward**
Mato Grosso do Sul	0.77	0.78	1.46	3.17 (−3.71;10.54)	0.399	Stationary
Mato Grosso	0.37	0.58	1.06	5.27 (−1.30;12.27)	0.153	Stationary
Goiás	0.47	0.69	1.13	5.90 (−0.71;12.96)	0.115	Stationary
Distrito Federal	0.51	0.52	0.92	2.68 (−2.02;7.61)	0.297	Stationary

Abbreviations – HRs: Hospitalization Rates; APC: Annual Percentage Change; 95%CI: 95% Confidence Interval.

**Table 2:** Mean and trend of length of stay and hospitalization rate for renal neoplasia in the SUS by sex and age group, per 10,000 inhabitants, Brazil, 2013–2023

Incidence rate by characteristics	HRs 2013	HRs 2023	Average HRs	APC (95%CI)	p-value	Trend
**IR by sex**						
Female IR	0.39	2.27	1.12	9.80 (4.49;15.37)	0.005	Upward
Male IR	0.59	2.97	1.55	8.85 (4.16;13.75)	0.004	Upward
**IR by age group**						
**Children and Adolescents**	**0.39**	**0.50**	**w0.43**	**0.82 (0.45;1.19)**	**0.002**	**Upward**
0 to 4 years	0.06	0.44	0.24	11.03 (6.34;15.92)	0.001	Upward
5 to 9 years	0.64	3.57	1.86	9.41 (5.20;13.78)	0.002	Upward
10 to 14 years	1.80	9.73	5.15	9.57 (4.73;14.63)	0.003	Upward
15 to 19 years	1.30	1.32	1.26	−0.27 (−0.68;0.14)	0.224	Stationary
**Young Adults**	**0.25**	**0.50**	**0.38**	**1.33 (0.51;2.16)**	**0.011**	**Upward**
20 to 24 years	0.07	0.12	0.09	4.30 (2.86;5.76)	<0.001	Upward
25 to 29 years	0.04	0.07	0.06	5.87 (2.50;9.36)	0.007	Upward
30 to 34 years	0.03	0.16	0.10	8.78 (4.70;13.03)	0.002	Upward
35 to 39 years	0.02	0.27	0.14	13.61 (10.08;17.25)	<0.001	Upward
**Adults**	**0.08**	**0.41**	**0.25**	**10.03 (3.98;16.43)**	**0.009**	**Upward**
40 to 44 years	0.12	0.89	0.48	10.93 (5.53;16.60)	0.003	Upward
45 to 49 years	0.24	1.82	0.86	11.28 (7.26;15.44)	0.000	Upward
50 to 54 years	0.39	2.66	1.42	10.64 (6.43;15.03)	0.001	Upward
55 to 59 years	0.84	4.42	2.30	8.96 (4.32;13.80)	0.004	Upward
**Young Elderly**	**1.31**	**6.18**	**3.31**	**8.69 (4.75;12.77)**	**0.002**	**Upward**
60 to 64 years	1.80	8.05	4.55	8.56 (3.87;13.45)	0.005	Upward
65 to 69 years	1.9	10.57	5.52	9.76 (4.91;14.82)	0.003	Upward
70 to 74 years	1.6	11.59	5.71	10.98 (6.39;15.77)	0.001	Upward
75 to 79 years	1.89	9.49	5.13	9.48 (3.46;15.85)	0.012	Upward
**Elderly 80+ (long-lived)**	**0.81**	**5.59**	**2.51**	**10.51 (5.84;15.38)**	**0.001**	**Upward**

Abbreviations – HRs: Hospitalization Rates; APC: Annual Percentage Change; 95%CI: 95% Confidence Interval.

The South (2.33/100,000) and Southeast (1.44/100,000) regions were notable for having mean HRs above the national average. An upward trend was observed across all regions of Brazil, with emphasis on the Southeast (APC: 9.62; 95%CI: 5.28 to 14.14; p = 0.002) and South (APC: 9.15; 95%CI: 4.99 to 13.48; p = 0.002), which recorded the highest percentage variations.

**Figure 1:**
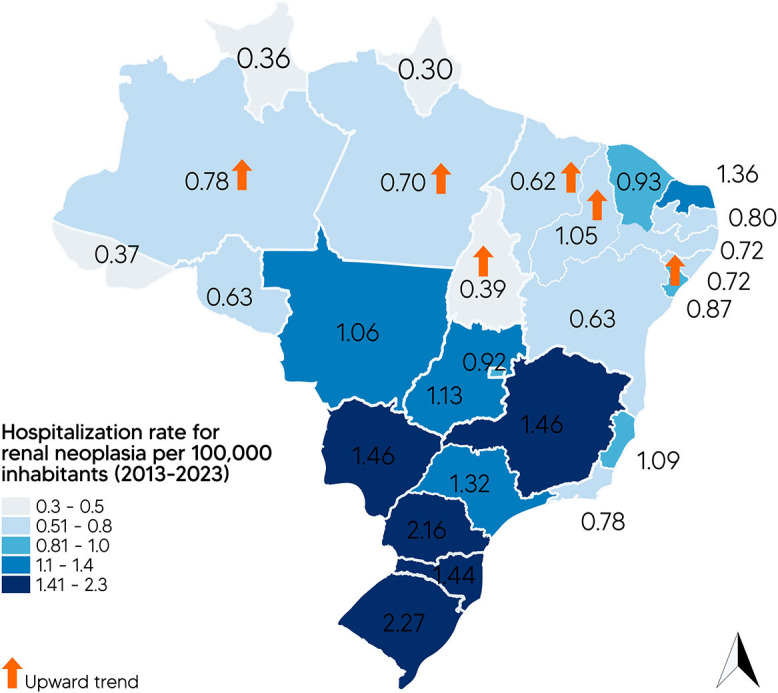
Map of the spatial distribution of mean Hospitalization Rates (HRs) of Renal Neoplasia (per 100,000 inhabitants) by State and indication of an upward trend for the period 2013 to 2023.

Source: Hospital Information System, 2023. Prepared by the authors.

Among the 27 states (UFs), most showed a stationary trend, except for the states of Amazonas (APC: 6.56; 95%CI: 1.60 to 11.76; p = 0.028), Pará (APC: 5.07; 95%CI: 1.29 to 8.99; p = 0.027), Tocantins (APC: 2.72; 95%CI: 1.32 to 4.15; p = 0.004), Maranhão (APC: 6.06; 95%CI: 3.25 to 8.95; p = 0.002), Piauí (APC: 4.95; 95%CI: 1.83 to 8.17; p = 0.012), and Sergipe (APC: 5.89; 95%CI: 2.40 to 9.50; p = 0.009), which showed significant growth. The states of Rio Grande do Sul (mean HR: 2.27/100,000) and Paraná (mean HR: 2.16/100,000) recorded the highest mean HRs (Figure 1).

The age group analysis revealed an upward trend in HRs in almost all age groups throughout the study period, ranging from children and adolescents to the long-lived elderly. Only the 15–19 age group exhibited a stationary trend. The highest mean HRs were observed among the young elderly (3.31/100,000) and the long-lived elderly individuals (2.51/100,000).

## Discussion

There has been a significant upward trend in HRs due to renal neoplasia in Brazil and its regions over the last decade. This substantial increase may be associated with the prevalence of risk factors for the disease, such as smoking, systemic arterial hypertension, and obesity^15^. In addition, improved access to healthcare services and advancements in diagnostic methods may have contributed to earlier and more frequent identification of these cases^16^. Especially for diseases in which the advanced stages have historically been poorly understood, these improvements are reflected in higher incidence rates^17^.

This epidemiological scenario in Brazil reflects the rapidly increasing global trend in the incidence of kidney cancer, one of the fastest-growing malignant neoplasms in terms of newly diagnosed cases^18^. The highest incidence of the disease is observed in Western countries, especially in the United States of America (USA), Canada, and European nations^19^. In the USA, for example, this neoplasm ranks as the ninth most common type of cancer. This geographic distribution could be partly explained by the greater availability of healthcare and technologies for early diagnosis^20^.

Regionally, the South and Southeast regions had the highest HRs for the studied period, exceeding the national average. This scenario may be related to better socioeconomic and development conditions^21^, as well as higher human development indices in these regions. Additionally, easier access to healthcare services and the higher prevalence of risk factors in urbanized and economically developed areas may explain the higher incidence of HRs in these regions^22^. As indicated by Motzer et al.^23^, the highest rates of renal cell carcinoma have been identified in European and North American countries, and to a lesser extent in Asian or South American countries. The authors attribute the variations in incidence rates in part to access to healthcare and diagnostic methods. The study by Ferreira et al.^24^ shows that the incidence of neoplasms in men in Brazil is more prevalent in populations with lower social vulnerability, predominantly in more developed areas, which may explain the higher incidence of renal neoplasia in the southern and southeastern regions.

Furthermore, the high rates in these regions may be related to the higher prevalence of risk factors for Chronic Non-Communicable Diseases (CNCD), particularly smoking and inadequate diet, according to cross-sectional literary evidence^25^. Although the causes of renal neoplasia are uncertain, there is a consensus that smoking is a significant risk factor for the development of the disease^26^. Thus, the high incidence of renal neoplasia in the Southern region may be related to the higher prevalence of smoking in this area, which accounts for 15.7% of the population^27^.

The analysis by sex revealed that, although both sexes showed a significant upward trend in HRs, the mean coefficients were higher in males compared to females. This finding is consistent with existing literature, which indicates a higher occurrence of renal neoplasia in men, possibly due to biological, hormonal, and behavioral differences, as they are the group most prone to developing such risk behaviors^28^.

Regarding smoking, evidence suggests a positive correlation between time abstaining from tobacco and a reduction in renal neoplasia incidence, reinforcing the role of tobacco as an important risk factor for the development of this neoplasm^29^. The risk is directly associated with tobacco use: male smokers have an approximately 50% higher risk of developing the neoplasm compared to never smokers, while in women the increase is approximately 20%, varying proportionally with the number of cigarettes consumed over time. In addition, studies indicate that after more than 10 years of smoking cessation, the risk associated with developing renal neoplasia decreases considerably, although it does not return to the levels of never smokers^30^.

Kidney cancer is more commonly diagnosed in the elderly population, with a mean age of 64 years in the USA, ranging from 65 to 74 years^31^. The data from the study expand on the global epidemiology, showing a higher occurrence of the disease in Brazil among patients aged 70 to 74, i.e., at an older age compared to the USA average. These data reinforce the predominance of the disease associated with an ageing population, revealing the impact of longevity on the incidence of neoplasms^32^ and the need to direct preventive strategies towards this population group.

Renal neoplasia is often identified asymptomati-cally and incidentally during the investigation of other conditions, often at more advanced stages^24^. Diagnosis usually occurs during the sixth and eighth decades of life, with very low incidences of cases diagnosed before the age of 40^15^. Cortez’s study highlights that more than 60% of kidney tumors are detected incidentally through imaging tests performed for other conditions, especially in the sixth and seventh decades of life^16^. This evidence underlines the need to strengthen early detection strategies to reduce the morbidity and mortality associated with renal neoplasia.

The nature of this study and the use of secondary data have important limitations. Underreporting and inadequate completion of information system collection tools compromise the accuracy of epidemiological analysis, which may lead to underestimation or overestimation of HRs. The lack of data prevents a more in-depth investigation of renal neoplasia, hindering analysis of the influence of individual risk factors, such as lifestyle habits, on the incidence and mortality associated with this disease.

Furthermore, the results indicated a significant increase in HRs for renal neoplasia in the South and Southeast regions. However, no statistically significant increase was observed in the individual analysis by state within these regions. This finding could be attributed to the methodological approach used for time series analysis in the study, namely, generalized linear regression using the Prais-Winsten method, which considers the data autocorrelation and allows for a global perspective on regional trends^19^. In aggregated analyses, such as at the regional level, the greater volume of data allows for the detection of trends that may not be perceptible in smaller samples, such as those at the state level. This regional profile tends to provide a broader view of healthcare services and the presence of specialized centers that are characteristic of each region, while in the states, local particularities become more evident.

However, this study significantly contributes to the epidemiology of renal neoplasia in Brazil. This is an unprecedented nationwide study analyzing temporal and regional trends in HRs, highlighting its relevance to public health in light of the paucity of literature on the disease. The results demonstrate the importance of strengthening public health policies aimed at prevention, early diagnosis, and treatment of renal neoplasia, while addressing individual risk factors is essential to tackle the growing burden of this disease in the country.

## Conclusion

There was a significant upward trend in HRs due to renal neoplasia in Brazil from 2013 to 2023, with regional variations and a predominance in males and in the over-60 age group.

## Data Availability

This study was based solely on public data made available by the Brazilian Ministry of Health, and did not require submission to and approval by the Research Ethics Committee. The study complies with the guidelines established by Resolution No. 510 of 2016 of the Brazilian National Health Council and is in accordance with Law No. 12,527 of November 18, 2011.
