# Supplementary material for: Epidemiological profile of kidney cancer in Brazil: a multiregional ecological study
Source: J Bras Nefrol. 2025 Mar 10;47(2):e20240180. doi: 10.1590/2175-8239-JBN-2024-0180en (PMC11913370; doi:10.1590/2175-8239-JBN-2024-0180en)
Supplement: Translation

# Translation: Perfil epidemiológico da neoplasia renal no Brasil: um estudo ecológico multirregional

**DOI:** 10.1590/2175-8239-JBN-2024-0180pt

**Authors:** Carlos Eduardo da Silva, Yasmin de Souza Ciriaco, Gustavo Machado Ribeiro, Laura Almeida Vidal, Verônica Aparecida Silva Cintra, Sabrina Thalita dos Reis

**Affiliations:** 1 Faculdade de Medicina Atenas Passos, Passos, MG, Brasil.; 2 Universidade de São Paulo, Faculdade de Medicina, Hospital das Clínicas, Laboratório de Investigação Médica 55, São Paulo, SP, Brasil.

**Keywords:** Neoplasias Renais, Epidemiologia, Hospitalização, Estudos de Séries Temporais

## Abstract

**Introdução:**

A neoplasia renal é uma doença complexa e heterogênea, caracterizada por alta morbimortalidade.

**Objetivo:**

Analisar a tendência temporal das taxas de internação (TIs) por neoplasia renal no Brasil, segmentadas por região, Unidades da Federação (UFs) e características populacionais, no período de 2013 a 2023.

**Métodos:**

Estudo ecológico utilizando dados do Sistema de Informações Hospitalares, pela análise das Autorizações de Internação Hospitalar, abrangendo o período de 2013 a 2023. A tendência anual das TIs foi analisada por meio de regressão linear generalizada pelo método Prais-Winsten pelo cálculo da Variação Percentual Anual (VPA), considerando sexo, idade, raça/cor e região/UF. Para as análises, adotou-se uma significância de 5%.

**Resultados:**

Foram registradas 31.388 hospitalizações por neoplasia renal no Brasil durante o período, evidenciando uma tendência crescente significativa nas TIs (VPA: 9,12; IC95%: 5,30; 13,1; p < 0,001). O aumento foi observado em ambos os sexos e em todas as regiões. Entre as UFs, a maioria apresentou tendências estacionárias. As maiores TIs médias foram identificadas entre idosos jovens (3,31/100 mil) e idosos longevos (2,51/100 mil).

**ConclusÃO:**

As TIs por neoplasia renal no Brasil apresentaram uma tendência crescente significativa entre 2013 e 2023, com variações regionais, predominância no sexo masculino e maior incidência na faixa etária acima de 60 anos.

## Introdução

A neoplasia renal é uma doença complexa e heterogênea, composta por subtipos tumorais que acometem os rins, cada qual com alterações histológicas, mutações genéticas, características clínicas e respostas ao tratamento distintas^1,2^. Dentre esses subtipos, o carcinoma de células renais (CCR) é o mais comum e representa aproximadamente 90% de todos os tumores renais, com destaque para o CCR de células claras (70% dos casos), CCR papilar (10 a 15%) e CCR cromófobo (5%)^3^.

As neoplasias renais são mais incidentes em homens do que em mulheres^4^, com idade média de diagnóstico aos 65 anos, e estão fortemente associadas a fatores de risco como tabagismo, obesidade, hipertensão arterial sistêmica e síndromes genéticas, como a síndrome de von Hippel-Lindau^5,6,7^. No âmbito epidemiológico, dados do Sistema de Informação sobre Mortalidade (SIM) registraram 18.306 óbitos por neoplasia renal no Brasil entre 2018 e 2022^8^. Esse cenário reflete um processo de envelhecimento populacional, associado à prevalência de fatores de risco para a doença, o que evidencia a relevância desse agravo de saúde pública^7^.

A neoplasia renal é inicialmente assintomática, mas pode se manifestar com sintomas inespecíficos, como dor em flancos, hematúria, massa abdominal palpável, febre e anemia, o que dificulta o diagnóstico precoce^9^. Por essa razão, a doença é frequentemente descoberta de forma incidental^10^, por meio de exames como ultrassom, ressonância ou tomografia abdominal, sendo posteriormente confirmada por biópsia para a classificação do tipo histopatológico: carcinoma renal de células claras (o mais incidente e com pior prognóstico), papilar (dividido microscopicamente em tipo 1, com melhor prognóstico, ou tipo 2) e cromófobo, que tem o melhor prognóstico quando comparado ao de células claras^11^.

Essa neoplasia é caracterizada por sua elevada taxa de mortalidade, principalmente devido ao seu início assintomático, à carência de rastreamento e à dificuldade em realizar o diagnóstico precoce, fatores que contribuem para o pior prognóstico e para a morte do paciente em decorrência da progressão da doença^10,12,13^. Dada a crescente relevância da neoplasia renal no cenário epidemiológico nacional, o mapeamento da distribuição regional e dos padrões de internações ao longo do tempo é relevante. Diante desse cenário desafiador, o presente estudo objetiva preencher essa lacuna, contribuindo com um mapeamento da epidemiologia da neoplasia renal no Brasil, com foco em sua distribuição geográfica, perfil sociodemográfico dos pacientes e tendências temporais.

## Métodos

Estudo ecológico, observacional, de abordagem quantitativa, com a análise das taxas de internações (TIs) por causa básica de neoplasia renal, entre os anos de 2013 a 2023, no Brasil, regiões e Unidades Federativas (UFs). Foram analisadas as Autorizações de Internação Hospitalar (AIHs) e estudadas as variáveis sexo, faixa etária, região brasileira e UF.

Os dados foram coletados no Departamento de Informática do Sistema Único de Saúde (DATASUS), utilizando-se o Sistema de Informação Hospitalar (SIH), por meio das AIHs. A AIH é um instrumento imprescindível para a consolidação das informações acerca das internações realizadas no âmbito do Sistema Único de Saúde, incluindo informações pormenorizadas dos atendimentos hospitalares, tais como diagnósticos, procedimentos, tempo de permanência, entre outras variáveis de interesse para a análise epidemiológica e assistencial. A causa básica foi definida pela classificação C64 da 10ª revisão da Classificação Internacional de Doenças (CID-10), referente à neoplasia maligna do rim, exceto a pelve renal.

Os dados foram extraídos e tabulados no Microsoft Excel® 2016 e, posteriormente, as variáveis quantitativas categóricas foram analisadas em frequências absolutas e relativas, sendo apresentadas por meio de tabelas e gráficos. As TIs anuais por neoplasia renal foram determinadas a partir de estimativas populacionais do Instituto Brasileiro de Geografia e Estatística (IBGE) para o período analisado, sendo calculadas pela razão entre o número de casos e o tamanho da população residente em cada região brasileira, multiplicado por 100 mil habitantes. As TIs anuais foram estratificadas por sexo (masculino e feminino), grupo etário (crianças e adolescentes, até 19 anos; adulto jovem, 20 a 39 anos; adulto, 40 a 59 anos; idoso jovem, 60 a 79 anos; idoso longevo, 80 anos ou mais) e região/UF (Norte [Rondônia, Acre, Amazonas, Roraima, Pará, Amapá e Tocantins], Nordeste [Maranhão, Piauí, Ceará, Rio Grande do Norte, Paraíba, Pernambuco, Alagoas, Sergipe e Bahia], Sudeste [São Paulo, Rio de Janeiro, Minas Gerais e Espírito Santo], Sul [Paraná, Santa Catarina e Rio Grande do Sul] e Centro-Oeste [Mato Grosso do Sul, Mato Grosso, Goiás e Distrito Federal]). A representação gráfica da neoplasia nas diferentes regiões brasileiras foi construída utilizando o software Quantum GIS (QGIS), versão 3.0.

Foi utilizada a regressão linear generalizada pelo método Prais-Winsten para a análise da tendência das TIs, conforme a descrição de Antunes e Cardoso^14^. A variação percentual anual (VPA) dos coeficientes foi calculada por meio do logaritmo dos coeficientes como variável dependente e os anos da série histórica como variável independente, adotando-se o número de anos da série –1. A tendência foi quantificada pela equação: VPA = [–1 + 10^b] * 100%, onde “b” indica a taxa de crescimento anual. Os intervalos de confiança (IC95%) foram calculados com base na fórmula: IC95% = [–1 + 10^b±t*se] * 100%. Os valores de “b” e o erro padrão (se) foram extraídos da análise de regressão, e o valor de “t” foi obtido da distribuição t de Student. Adotou-se uma significância de 5% (p < 0,05), considerando a tendência crescente quando a taxa foi positiva, decrescente quando negativa e estacionário quando não houve diferença estatisticamente significativa (p > 0,05). A análise foi realizada utilizando o *software R Project for Statistical Computing*, versão 3.6.0, com o auxílio do Excel 2016 para construção do banco de dados.

Este estudo se baseou exclusivamente em dados públicos disponibilizados pelo Ministério da Saúde, não sendo necessária a aprovação pelo Comitê de Ética em Pesquisa. O estudo segue as diretrizes estabelecidas pela Resolução n.º 510, de 2016, do Conselho Nacional de Saúde, e está de acordo com a Lei n.º 12.527, de 18 de novembro de 2011.

## Resultados

Entre 2013 e 2023, foram registradas 31.388 hospitalizações por neoplasia renal no Brasil. Durante esse período, observou-se uma tendência crescente significativa nas TIs, que passaram de 0,48 por 100 mil habitantes em 2013 para 2,62 por 100 mil habitantes em 2023, com uma TI média de 1,35 por 100 mil habitantes (VPA: 9,12; IC95%: 5,30; 13,1; p < 0,001) (Tabela 1). Tanto no sexo masculino quanto no feminino, as TIs apresentaram tendência significativa de crescimento, com coeficientes médios de 1,55 e 1,12 por 100 mil habitantes, respectivamente (Tabela 2).

**Tabela 1:** Média e tendência do tempo de permanência e taxa de internação por neoplasia renal no SUS segundo região e Unidade da Federação (UF), Brasil, 2013–2023

Região e UF	TI 2013	TI 2023	TI Média	VPA (IC95%)	p-valor	Tendência
**Brasil**	**0,48**	**2,62**	**1,35**	**9,12 (5,30;13,1)**	**< 0,001**	**Crescente**
**Norte**	**0,30**	**1,40**	**0,73**	**7,61 (6,2;9,05)**	**< 0,001**	**Crescente**
Rondônia	0,18	0,29	0,63	4,95 (−0,35;10,55)	0,101	Estacionária
Acre	0,75	0,25	0,37	3,42 (−5,28;12,95)	0,472	Estacionária
Amazonas	0,35	0,39	0,78	6,56 (1,60;11,76)	0,028	Crescente
Roraima	0,00	0,26	0,36	3,28 (−8,42;16,47)	0,612	Estacionária
Pará	0,27	0,38	0,70	5,07 (1,29;8,99)	0,027	Crescente
Amapá	0,54	0,23	0,30	−2,82 (−9,94;4,86)	0,480	Estacionária
Tocantins	0,20	0,24	0,39	2,72 (1,32;4,15)	0,004	Crescente
**Nordeste**	**0,37**	**1,80**	**0,91**	**8,56 (5,49;11,71)**	**< 0,001**	**Crescente**
Maranhão	0,32	0,34	0,62	6,06 (3,25;8,95)	0,002	Crescente
Piauí	0,62	0,61	1,05	4,95 (1,83;8,17)	0,012	Crescente
Ceará	0,40	0,52	0,93	5,33 (−1,19;12,28)	0,146	Estacionária
Rio Grande do Norte	0,42	0,68	1,36	7,02 (0,63;13,80)	0,059	Estacionária
Paraíba	0,39	0,43	0,80	4,14 (−1,11;9,66)	0,158	Estacionária
Pernambuco	0,47	0,42	0,72	3,26 (−1,29;8,02)	0,196	Estacionária
Alagoas	0,34	0,36	0,72	6,51 (−2,31;16,11)	0,186	Estacionária
Sergipe	0,23	0,49	0,87	5,89 (2,40;9,50)	0,009	Crescente
Bahia	0,26	0,30	0,63	4,26 (−3,22;12,32)	0,300	Estacionária
**Sudeste**	**0,48**	**2,83**	**1,44**	**9,62 (5,28;14,14)**	**0,002**	**Crescente**
Minas Gerais	0,47	0,69	1,46	5,30 (−2,84;14,12)	0,240	Estacionária
Espírito Santo	0,54	0,58	1,09	5,26 (−0,94;11,85)	0,132	Estacionária
Rio de Janeiro	0,43	0,39	0,78	3,58 (−1,87;9,34)	0,234	Estacionária
São Paulo	0,50	0,66	1,32	4,53 (−3,47;13,19)	0,304	Estacionária
**Sul**	**0,82**	**4,38**	**2,33**	**9,15 (4,99;13,48)**	**0,002**	**Crescente**
Paraná	0,96	1,12	2,16	3,91 (−2,99;11,31)	0,302	Estacionária
Santa Catarina	0,48	0,68	1,44	4,93 (−2,27;12,66)	0,217	Estacionária
Rio Grande do Sul	0,88	1,19	2,27	4,97 (−2,58;13,11)	0,235	Estacionária
**Centro-Oeste**	**0,51**	**2,47**	**1,30**	**8,42 (4,34;12,67)**	**0,003**	**Crescente**
Mato Grosso do Sul	0,77	0,78	1,46	3,17 (−3,71;10,54)	0,399	Estacionária
Mato Grosso	0,37	0,58	1,06	5,27 (−1,30;12,27)	0,153	Estacionária
Goiás	0,47	0,69	1,13	5,90 (−0,71;12,96)	0,115	Estacionária
Distrito Federal	0,51	0,52	0,92	2,68 (−2,02;7,61)	0,297	Estacionária

Abreviaturas – TI: Taxa de Internação; VPA: Variação Percentual Anual; IC95%: Intervalo de Confiança 95%.

**Tabela 2:** Média e tendência do tempo de permanência e taxa de internação por neoplasia renal no SUS por sexo e faixa etária, por 10 mil habitantes, Brasil, 2013–2023

TI segundo características	TI 2013	TI 2023	TI Média	VPA (IC95%)	p-valor	Tendência
**TI por sexo**						
TI feminina	0,39	2,27	1,12	9,80 (4,49;15,37)	0,005	Crescente
TI masculina	0,59	2,97	1,55	8,85 (4,16;13.75)	0,004	Crescente
**TI por faixa etária**						
**Crianças e adolescentes**	**0,39**	**0,50**	**0,43**	**0,82 (0,45;1,19)**	**0,002**	**Crescente**
0 até 4 anos	0,06	0,44	0,24	11,03 (6,34;15,92)	0,001	Crescente
5 a 9 anos	0,64	3,57	1,86	9,41 (5,20;13,78)	0,002	Crescente
10 a 14 anos	1,80	9,73	5,15	9,57 (4,73;14,63)	0,003	Crescente
15 a 19 anos	1,30	1,32	1,26	−0,27 (−0,68;0,14)	0,224	Estacionária
**Adulto jovem**	**0,25**	**0,50**	**0,38**	**1,33 (0,51;2,16)**	**0,011**	**Crescente**
20 a 24 anos	0,07	0,12	0,09	4,30 (2,86;5,76)	< 0,001	Crescente
25 a 29 anos	0,04	0,07	0,06	5,87 (2,50;9,36)	0,007	Crescente
30 a 34 anos	0,03	0,16	0,10	8,78 (4,70;13,03)	0,002	Crescente
35 a 39 anos	0,02	0,27	0,14	13,61 (10,08;17,25)	< 0,001	Crescente
**Adulto**	**0,08**	**0,41**	**0,25**	**10,03 (3,98;16,43)**	**0,009**	**Crescente**
40 a 44 anos	0,12	0,89	0,48	10,93 (5,53;16,60)	0,003	Crescente
45 a 49 anos	0,24	1,82	0,86	11,28 (7,26;15,44)	0,000	Crescente
50 a 54 anos	0,39	2,66	1,42	10,64 (6,43;15,03)	0,001	Crescente
55 a 59 anos	0,84	4,42	2,30	8,96 (4,32;13,80)	0,004	Crescente
**Idoso jovem**	**1,31**	**6,18**	**3,31**	**8,69 (4,75;12,77)**	**0,002**	**Crescente**
60 a 64 anos	1,80	8,05	4,55	8,56 (3,87;13,45)	0,005	Crescente
65 a 69 anos	1,9	10,57	5,52	9,76 (4,91;14,82)	0,003	Crescente
70 a 74 anos	1,6	11,59	5,71	10,98 (6,39;15,77)	0,001	Crescente
75 a 79 anos	1,89	9,49	5,13	9,48 (3,46;15,85)	0,012	Crescente
**Idosos 80+ (longevo)**	**0,81**	**5,59**	**2,51**	**10,51 (5,84;15,38)**	**0,001**	**Crescente**

Abreviaturas – TI: Taxa de Internação; VPA: Variação Percentual Anual; IC95%: Intervalo de Confiança 95%.

As regiões Sul (2,33/100 mil) e Sudeste (1,44/100 mil) destacaram-se por apresentarem TIs médias acima da média nacional. Observou-se uma tendência crescente em todas as regiões do Brasil, com ênfase no Sudeste (VPA: 9,62; IC95%: 5,28 a 14,14; p = 0,002) e no Sul (VPA: 9,15; IC95%: 4,99 a 13,48; p = 0,002), que registraram as maiores variações percentuais.

Entre as 27 UFs, a maioria apresentou tendência estacionária, com exceção dos estados do Amazonas (VPA: 6,56; IC95%: 1,60 a 11,76; p = 0,028), Pará (VPA: 5,07; IC95%: 1,29 a 8,99; p = 0,027), Tocantins (VPA: 2,72; IC95%: 1,32 a 4,15; p = 0,004), Maranhão (VPA: 6,06; IC95%: 3,25 a 8,95; p = 0,002), Piauí (VPA: 4,95; IC95%: 1,83 a 8,17; p = 0,012) e Sergipe (VPA: 5,89; IC95%: 2,40 a 9,50; p = 0,009), que exibiram crescimento significativo. Os estados do Rio Grande do Sul (TI média: 2,27/100 mil) e Paraná (TI média: 2,16/100 mil) registraram as maiores TIs médias (Figura 1).

**Figura 1:** Mapa da distribuição espacial das TIs médias de neoplasia renal (por 100 mil habitantes) por Unidade da Federação e indicativo de tendência crescente para o período 2013 a 2023.

Fonte: Sistema de Informações Hospitalares, 2023. Elaborado pelos autores.

A análise por faixa etária revelou uma tendência de aumento das TIs em quase todos os grupos etários ao longo do período estudado, desde crianças e adolescentes até idosos longevos. Apenas a faixa etária de 15 a 19 anos apresentou tendência estacionária. As maiores TIs médias foram observadas entre idosos jovens (3,31/100 mil) e idosos longevos (2,51/100 mil).

## Discussão

Houve uma tendência significativa de aumento nas TIs por neoplasia renal no Brasil e em suas regiões ao longo da última década. Esse crescimento substancial pode estar associado à prevalência de fatores de risco para a doença, como tabagismo, hipertensão arterial sistêmica e obesidade^15^. Além disso, a melhoria no acesso aos serviços de saúde e o avanço nos métodos diagnósticos podem ter contribuído para a identificação mais precoce e frequente desses casos^16^. Especialmente para doenças cujos estágios avançados são historicamente mal compreendidos, essas melhorias refletem-se em taxas de incidência mais elevadas^17^.

Esse cenário epidemiológico no Brasil reflete a rápida tendência global de aumento da incidência do câncer renal, uma das neoplasias malignas com maior crescimento em novos casos diagnosticados^18^. A maior incidência da doença é observada em países ocidentais, especialmente nos Estados Unidos da América (EUA), Canadá e em nações europeias^19^. Nos EUA, por exemplo, essa neoplasia corresponde ao nono tipo de câncer mais comum. Essa distribuição geográfica pode ser parcialmente explicada pela maior disponibilidade de cuidados de saúde e de tecnologias para o diagnóstico precoce^20^.

Regionalmente, as Regiões Sul e Sudeste apresentaram as maiores TIs para o período estudado, ultrapassando a média nacional. Esse cenário pode estar relacionado às melhores condições socioeconômicas e de desenvolvimento^21^, além dos maiores índices de desenvolvimento humano nessas regiões. Além disso, a facilidade de acesso aos serviços de saúde e a maior prevalência de fatores de risco em áreas urbanizadas e economicamente desenvolvidas podem explicar a maior incidência de TIs nessas regiões^22^. Conforme apontado por Motzer et al.^23^, as maiores taxas de carcinoma de células renais são identificadas em países da Europa e da América do Norte, sendo identificadas em menor proporção em países asiáticos ou da América do Sul. Os autores relacionam as variações nas taxas de incidência, em parte, ao acesso aos cuidados de saúde e a métodos diagnósticos. O estudo de Ferreira et al.^24^ indica que a incidência de neoplasias em homens no Brasil é mais prevalente em populações com menor vulnerabilidade social, predominante nas áreas mais desenvolvidas, o que pode justificar a maior incidência da neoplasia renal nas regiões Sul e Sudeste.

Ademais, as altas taxas nessas regiões podem estar relacionadas à maior prevalência de fatores de risco para Doenças Crônicas Não Transmissíveis (DCNT), especialmente o tabagismo e a alimentação inadequada, conforme evidências literárias transversais^25^. Embora as causas da neoplasia renal sejam incertas, há o consenso de que o hábito do tabagismo seja um fator de risco significativo para o desenvolvimento da doença^26^. Assim, a alta incidência da neoplasia renal na região Sul pode se relacionar com a maior prevalência de tabagismo nessa região, que corresponde a 15,7% da população^27^.

A análise por gênero revelou que, embora ambos os sexos tenham apresentado tendência de crescimento significativo nas TIs, os coeficientes médios foram mais elevados no sexo masculino em comparação ao sexo feminino. Esse achado está alinhado à literatura existente, que aponta maior ocorrência de neoplasia renal entre os homens, possivelmente devido a diferenças biológicas, hormonais e comportamentais, tendo em vista que constituem o grupo mais propenso a desenvolver tais comportamentos de risco^28^.

Em relação ao tabagismo, as evidências apontam uma correlação positiva entre o tempo de abstinência do tabaco e a diminuição na incidência de neoplasia renal, o que reforça o papel do tabaco como importante fator de risco para o desenvolvimento dessa neoplasia^29^. O risco está diretamente relacionado à carga tabágica: homens fumantes apresentam um risco cerca de 50% maior de desenvolver a neoplasia em comparação com nunca fumantes, enquanto nas mulheres o aumento é de aproximadamente 20%, variando proporcionalmente com o número de cigarros ao longo do tempo. Além disso, estudos indicam que, após mais de 10 anos de cessação do tabagismo, o risco associado ao desenvolvimento da neoplasia renal reduz consideravelmente, embora não retorne aos níveis dos nunca fumantes^30^.

O câncer renal é uma doença mais diagnosticada na população idosa, sendo a média nos EUA de 64 anos e variando entre 65 e 74 anos^31^. Os dados do estudo ampliam a epidemiologia mundial, evidenciando maior ocorrência da doença no Brasil entre pacientes na faixa etária de 70 a 74 anos, ou seja, em uma idade mais avançada em comparação com a média nos EUA. Esses dados reforçam a predominância da doença associada ao envelhecimento populacional, revelando o impacto da longevidade na incidência de neoplasias^32^ e a necessidade de estratégias preventivas direcionadas a esse grupo populacional.

A neoplasia renal é frequentemente identificada de forma assintomática e incidental durante a investigação de outras condições, muitas vezes já em estágios mais avançados^24^. O diagnóstico geralmente ocorre durante a sexta e a oitava décadas de vida, com incidências muito baixas de casos diagnosticados antes dos 40 anos^15^. O estudo de Cortez destaca que mais de 60% dos tumores renais são descobertos incidentalmente por exames de imagem realizados para outras condições, especialmente nas sextas e sétimas décadas de vida^16^. Essas evidências sublinham a necessidade de fortalecimento das estratégias de detecção precoce para reduzir a morbimortalidade associada à neoplasia renal.

A natureza deste estudo e a utilização de dados secundários apresentam limitações importantes. A subnotificação e o preenchimento inadequado das ferramentas de coleta dos sistemas de informação comprometem a precisão da análise epidemiológica, podendo levar à subestimação ou superestimação das TIs. A ausência de dados impede uma investigação mais aprofundada da neoplasia renal, impedindo a análise da influência dos fatores individuais de risco, como os hábitos de vida, na incidência e mortalidade por essa doença.

Ademais, os resultados indicaram um aumento significativo nas TIs por neoplasia renal nas regiões Sul e Sudeste, porém sem aumento estatisticamente significativo na análise individual por estado nessas regiões. Esse achado pode ser atribuído à abordagem metodológica de análise de séries temporais utilizada no estudo, a saber, a regressão linear generalizada pelo método de Prais-Winsten, que considera a autocorrelação dos dados e permite uma perspectiva global das tendências regionais^19^. Em análises agregadas, como no caso das regiões, o maior volume de dados permite captar tendências que podem não ser perceptíveis em amostras menores, como as dos estados. Esse perfil regional tende a fornecer uma visão mais ampla dos serviços de saúde e da presença de centros especializados característicos de cada região, enquanto nos estados, as particularidades locais se tornam mais evidentes.

Entretanto, este estudo contribui significativamente para a epidemiologia da neoplasia renal no Brasil. Trata-se de um estudo inédito, a nível nacional, com a análise das tendências temporais e regionais das TIs, o que evidencia sua relevância para a saúde pública pela escassez de informações na literatura sobre a doença. Os resultados demonstram a relevância do fortalecimento de políticas de saúde pública direcionadas às medidas de prevenção, diagnóstico precoce e tratamento da neoplasia renal, sendo a abordagem dos fatores de risco individuais essencial para enfrentar a crescente carga dessa doença no país.

## Conclusão

Houve tendência crescente significativa nas TIs por neoplasia renal no Brasil de 2013 a 2023, com variações regionais e predominância no sexo masculino e na faixa etária acima de 60 anos.

## Data Availability

Este estudo se baseou exclusivamente em dados públicos disponibilizados pelo Ministério da Saúde, não sendo necessária a apresentação e aprovação pelo Comitê de Ética em Pesquisa. O estudo segue as diretrizes estabelecidas pela Resolução n.º 510, de 2016, do Conselho Nacional de Saúde, e está de acordo com a Lei n.º 12.527, de 18 de novembro de 2011.
